# Open notebook science can maximize impact for rare disease projects

**DOI:** 10.1371/journal.pbio.3000120

**Published:** 2019-01-28

**Authors:** Rachel J. Harding

**Affiliations:** Structural Genomics Consortium, University of Toronto, Toronto, Canada

## Abstract

Transparency lies at the heart of the open lab notebook movement. Open notebook scientists publish laboratory experiments and findings in the public domain in real time, without restrictions or omissions. Research on rare diseases is especially amenable to the open notebook model because it can both increase scientific impact and serve as a mechanism to engage patient groups in the scientific process. Here, I outline and describe my own success with my open notebook project, LabScribbles, as well as other efforts included in the openlabnotebooks.org initiative.

## Abstract 2

This Research Matters article proposes that open notebooks present an opportunity for researchers to showcase their work, push the boundaries of open research practises and rapidly advance science in their field by making an immediate impact in their communities.

The term “open notebook science” was first coined in 2006 by Professor Jean-Claude Bradley, a Canadian chemistry researcher at Drexel University. In defining the practice, Bradley said, “It is essential that all of the information available to the researchers to make their conclusions is equally available to the rest of the world” [1], meaning that the notebook must be a complete and honest representation of the scientist’s findings. Despite the benefits of openly documenting research projects in real time, scientists have been slow to adopt open notebook science. Of those who have, many quickly abandon the practice or fail to update their notebook regularly or share it with restrictions.

Starting my own open notebook for my postdoctoral research project was appealing for a number of reasons. I am a postdoctoral fellow at the Structural Genomics Consortium (SGC), where open science is a critical part of the laboratory ethos. SGC scientists not only make their work as open as possible through extensive data and material sharing but have also recently implemented an open publication strategy, in which all manuscripts are submitted systematically to open access preprint servers. Piloting innovative open science strategies is well supported and is encouraged for scientists working within the SGC.

My particular research focus is Huntington disease (HD), a devastating inherited neurodegenerative disease. Although scientists mapped the causative mutation 25 years ago [2], successful development of disease-modifying or curative therapeutics has not materialized as hoped [3]. Open science and open notebooks promise to accelerate the process of scientific discovery. I hoped that by documenting my research project through an open notebook and sharing data ahead of traditional publication timelines, I would speed up the research process for HD (Fig 1). My specific aim was to create an open and collaborative network of researchers focused on answering some of the critical questions of HD protein biochemistry and reduce unnecessary duplication of effort.

**Fig 1:**
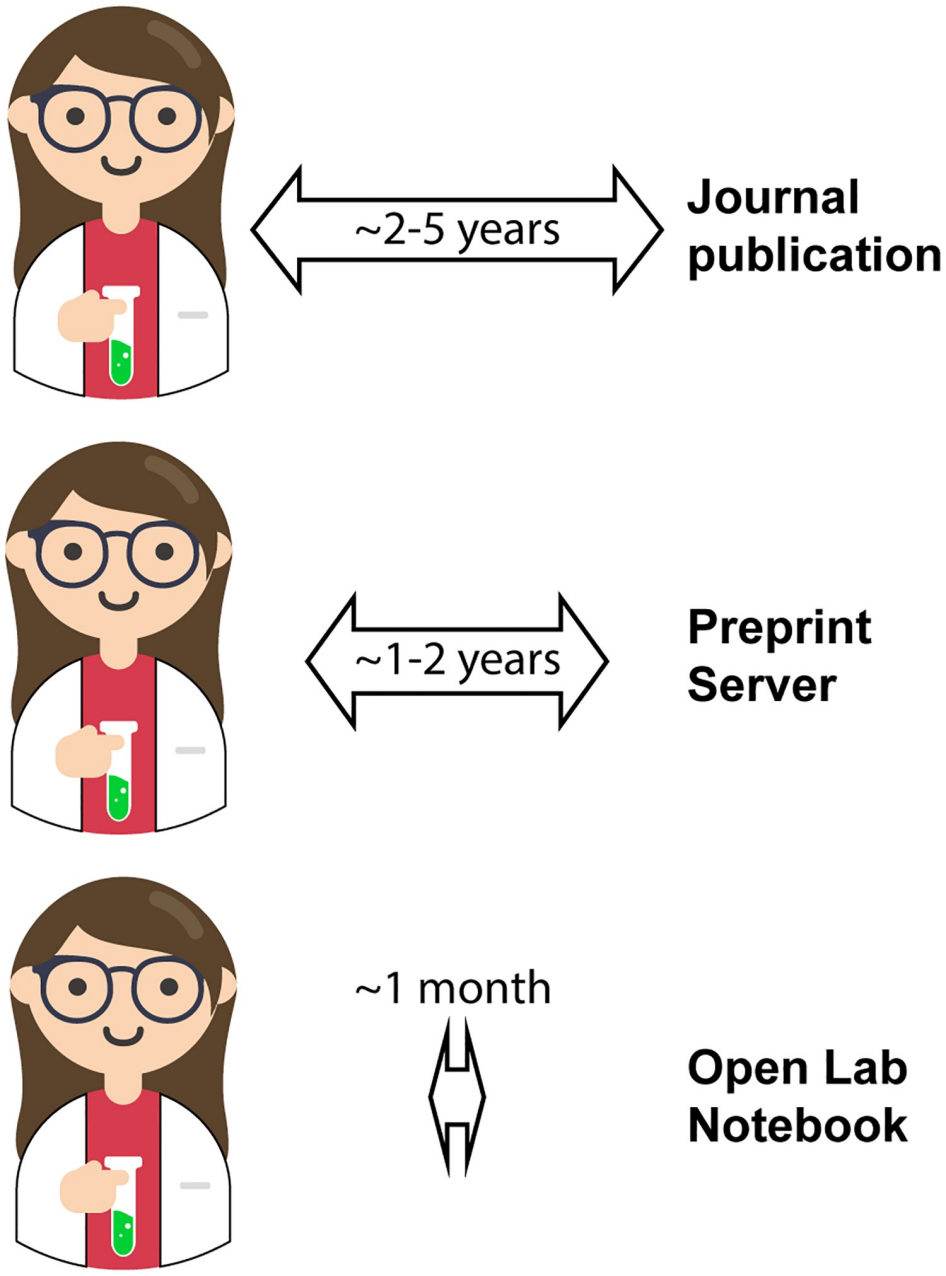
Open notebooks drastically reduce the time frame from bench to publication in the public domain. Image adapted from DataBase Center for Life Science (DBCLS), 201705 Scientist bench F. Image was adapted by Rachel J. Harding, CC BY 4.0.

The notebook format I have adopted is inspired by the groundwork laid by other open “notebookers”: it’s designed to be discoverable, accessible, clear, and detailed in its presentation, and to permit dialogue between readers and me, and to pave the way for collaborations. After I finish an experiment, I write up all the details of the materials and methods as well as all the data and analyses, ensure they’re in suitable file formats, and upload them to Zenodo as a data set licensed with Creative Commons Attribution 4.0. I then write a lay summary of the experiment on my LabScribbles blog, linking to the data set deposition, with the aim of reaching a wider nonspecialist audience. In this, my aim is to give context to this experiment within my broader research project, as well as provide guidance on points for improvement and next steps. Links to the Zenodo and blog post are then shared via a Twitter post.

Sharing my work via the open notebook has helped both my professional and personal development. The availability of my work has attracted collaborations with more than 10 international laboratories on my HD research. Data sets, materials, and instrument access are being freely shared back and forth, and I have received excellent feedback and advice from more senior researchers. Professor Ray Truant, an established HD researcher at McMaster University, is now one of my mentors for my Huntington’s Disease Society of America (HDSA) Berman-Topper Family career development fellowship. Professor Susan Lea, an expert in protein structural biology and director of the Central Oxford Structural Molecular Imaging Centre (COSMIC EM) facility at the University of Oxford, is a collaborator for electron microscopy work, work funded by a successful joint grant application to the Huntington Society of Canada. My writing and communications skills are also improving rapidly, particularly my ability to communicate with both scientific and general audiences. My professional network is also growing beyond the scientific sphere to include different stakeholder groups, including numerous HD patient organizations. I have also found that writing the notebook has improved my project planning skills and facilitated my progress through regular written reflection of my research.

Science is a competitive field, but I have been pleasantly surprised by the community’s response. Although many scientists might fear being scooped if they adopt open notebooks, my notebook has served as an opportunity to showcase my research project in the HD field, which has in turn protected me from being scooped by stamping my preliminary work and findings with a digital object identifier (DOI). The success of the project has subsequently led to generous funding support from HD research funding agencies, including the CHDI Foundation, the Huntington Society of Canada, and a career development fellowship from the Huntington’s Disease Society of America.

These successes, however, depend on commitment. The biomedical open notebook community has launched many efforts with enthusiasm only to lose steam and interest. In an effort to catalyze a sustainable community, earlier this year, the SGC launched a wider open notebook initiative with the inception of openlabnotebooks.org [4], a platform available to all scientists. A growing group of researchers from Canada, the United States, France, the United Kingdom, and Sweden are now running their own open notebooks. Building from the LabScribbles model, notebook data are deposited in Zenodo in the open notebook community, and lay summaries are written on the Open Lab Notebook blog site. Many of the projects are focused on rare diseases, often with funding from rare-disease patient-driven foundations. We believe rare-disease—driven projects are especially suitable for the open notebook format, because there are too few resources available and it is critical to make the most of every investment. Moreover, in a smaller research community, there is a greater chance to make an impact with an open notebook and for early career researchers to carve out a niche for themselves. Patient groups and advocacy organizations of these diseases truly appreciate these outreach efforts. Four of these open notebook projects are contributing to the scientific progress of the first open source pharmaceutical company, M4KPharma, which aims to discover and develop affordable drugs for those rare pediatric diseases that are not well served by current drug discovery business models. Monthly M4KPharma meetings are publicly broadcasted and archived online, where the work from these projects, including the company’s medicinal chemistry program, is discussed.

In an age when sharing daily aspects of our lives online through social media and other platforms is normalized, it’s a logical step to apply similar practices to scientific research. Open notebooks present an opportunity for researchers to showcase their work, push the boundaries of open research practices, and rapidly advance science in their field by making an immediate impact in their communities.

## Abbreviations

COSMIC EM: Central Oxford Structural Molecular Imaging Centre
DOI: digital object identifier
HD: Huntington disease
HDSA: Huntington’s Disease Society of America
SGC: Structural Genomics Consortium
